# Dental Caries Status among Yi Preschool Children in Yunnan Province, China: A Cross-Sectional Study

**DOI:** 10.3390/ijerph18168393

**Published:** 2021-08-08

**Authors:** Ni Zhou, Hui Ding, Juan Liu, Jieyi Chen, Shinan Zhang, Chun-Hung Chu

**Affiliations:** 1 School of Stomatology, Kunming Medical University, Kunming 650000, China; zhouni@kmmu.edu.cn (N.Z.); 20190236@kmmu.edu.cn (H.D.); liujuan@kmmu.edu.cn (J.L.); 2 Guanghua School of Stomatology, Sun Yat-sen University, Guangzhou 510000, China; chenjy679@mail.sysu.edu.cn; 3 Faculty of Dentistry, The University of Hong Kong, Hong Kong, China; chchu@hku.hk

**Keywords:** child, cross-sectional study, dental caries, oral health, ethnic group

## Abstract

Background: The Yi ethnic group is the sixth largest minority in China. The aim of this study was to investigate dental caries status among Yi preschool children in the Yunnan province, China. Method: This cross-sectional study invited 5-year-old Yi children using multistage cluster sampling. Two trained and calibrated dentists examined the children in kindergartens. The children’s dental caries experience was assessed using the decayed, missing, and filled teeth (dmft) index. Visual plaque on an indexed tooth of each six sextants was recorded and the oral hygiene status was assessed using the Visible Plaque Index. Parents completed questionnaires that surveyed their educational attainment. The questionnaires also collected information about the children’s demographics, snacking habits, oral-health-related behaviors and dental visit experiences. The associations between caries experiences and potential factors were analyzed using zero-inflated negative binomial regression. Results: All 452 invited children (249 boys, 55%) participated in this study with a response rate of 100%. Dental caries prevalence rate was 83%. The mean dmft score and decayed teeth score were 5.2 ± 4.4 and 5.1 ± 4.4, respectively. Almost half of the children (n = 211, 47%) had visible plaque on four or more of the six sextants. Most (n = 366, 81%) of the children had not visited a dentist in the prior 12 months. Regression analysis found the children’s caries experience was associated with their dental visit experience. Conclusion: The great majority of the Yi preschool children experienced dental caries and almost all of the cavities were not restored. Their oral hygiene was poor and visible plaque was commonly found on their teeth.

## 1. Introduction

China’s population is comprised of 56 ethnic groups. According to the data from the Tabulation on the population census of the People’s Republic of China, the Yi ethnic group is the sixth largest minority in China with a population of 8,714,393 [1]. Scattering in the Tibetan-Yi-Corridor and Wumeng Destitute Areas, the Yi nationality is also known as the ‘Lolo’ population, and they have a common ancestor with the Tibetan, Nakhi and Qiang peoples [2,3,4]. Located along the southwest border of China, Yunnan is China’s most diverse province, with 26 unique ethnic minorities living there for generations, and it holds the largest population of the Yi nationality [5]. Meanwhile, the Yi nationality is the largest minority group in Yunnan. A number of Yi autonomous areas exist in the Yunnan province.

Dental caries refers to “a biofilm-mediated, sugar-driven, multifactorial, dynamic disease that results in the phasic demineralization and remineralization of dental hard tissues” [6]. A meta-analysis performed based on 81 articles published from 1995 to 2019 indicated that the prevalence of dental caries in primary teeth was increased through the years [7]. The caries prevalence in primary teeth globally was estimated to be 46%, while in developing countries, the prevalence was higher [7,8]. An epidemiological study conducted in 31 regions in mainland China revealed the occurrence of dental caries among Yi adolescents was the second highest in the nation [9]. Additionally, a cross-sectional study conducted in 1999 reported that the decayed teeth were rarely restored among Yi children [10]. Uncontrolled dental caries can have a negative effect on children’s early development (e.g., reduced speech development, delayed growth curve, altered sleep patterns or eating habits, reduced ability to learn or perform usual activities) [11,12]. In addition, dental caries can have negative psychological outcomes for children and their caregivers (e.g., lower quality of life and poor self-esteem or emotional status) [11,12]. Prior studies which had been conducted in minority ethnic areas demonstrated that children’s caries experience was related to poor oral hygiene status, inappropriate snacking habits, dental attendance, or family socio-economic status [13,14]. It has been revealed that children with low socio-economic status showed a higher decayed, missing, and filled teeth (dmft) index [8]. Although the life quality of the Yi population has improved significantly during the past years, the economic development in Yi ethnic regions still lag behind. The GDP per capita of the Yi Autonomous Prefecture was around RMB 15,000 (USD 2000) in 2012, which was lower than the provincial GDP per capita [15]. Due to the economic burden and insufficient dental care facilities, the dental treatment needs were not properly met among Yi children [10].

To the best of our knowledge, no updated epidemiological data have revealed the oral health status among Yi preschool children or the effect of social determinants on their dental caries status. Determining the dental caries status and potential risk factors among young children is essential because the epidemiological data could form the basis for planning and implementing healthcare services to enhance children’s oral health status [14]. The World Health Organization (WHO) recommended that a 5-year age group could be employed as the index age group to investigate the dental caries status among preschool children [16]. Therefore, the present study was conducted to investigate the dental caries status among 5-year-old Yi children in Yunnan.

## 2. Materials and Methods

### 2.1. Study Design and Population

This cross-sectional study was conducted in 2016 and was reported in accordance with the Strengthening the Reporting of Observational Studies in Epidemiology guidelines. The Kunming Medical University Institutional Review Board reviewed and approved the study protocol, questionnaires, and consent forms before the commencement of participant recruitment. The target population was ethnic Yi preschool children residing in Yunnan. The inclusion criteria of the study population were (a) at the age of five, (b) Yi ethnicity and have lived in Yunnan since birth, (c) no intellectual or developmental disabilities, and (d) no long-term, systemic diseases. Children whose parents refused to sign the consent forms or children who could not cooperate with the dental examinations were excluded.

### 2.2. Sample Size Calculation and Sample Selection

Based on the preliminary findings of the prior studies, the prevalence of dental caries among Yi children was estimated to be 36% [17]. With the estimated 10% width of the 95% confidence interval, approximately 354 children were required according to the sample size calculation formula (n = 4 × 1.96^2^ × P × [1 − P]/L^2^, where n is the number of participants, P is the estimated prevalence rate of disease, and L is the width of the confidence interval which was 0.10 in this study) [18]. An 80% response rate was assumed; thus, at least 443 children should be initially recruited.

A multistage cluster sampling method was employed in the participant recruitment process. Among the 16 counties in the Yunnan province [14], most of the Yi ethnic minorities reside in the northern counties and some are scattered in the southeastern counties. We first chose the counties which were located in northern and south-eastern regions. Then, a list of kindergartens in these counties was obtained from the Yunnan Education Bureau. We numbered and selected kindergartens by a simple randomization method using a computer generated the random sequence. All 5-year-old Yi children in the selected kindergartens were invited until the required sample sizes were fulfilled.

### 2.3. Data Collection

A set of structured questionnaires were delivered to the parents or primary caregivers who accompanied the recruited children to the kindergartens before the dental examinations. The questionnaires covered two domains, namely, the children’s background information (sex, parents’ education attainment and household monthly income), and the children’s oral-health-related behaviors (tooth-brushing practices, snacking habits and dental visit experience) [19].

Children whose parents returned the questionnaires received a comprehensive dental examination in their kindergartens. Their dental caries status and oral hygiene status were examined by two trained and calibrated pediatric dentists by using CPI probes and a disposable dental mirror attached with a LED light. Dental caries was diagnosed following the World Health Organization criteria [16], which involves using the dmft index. Six index teeth (#55, #53, #51, #71, #73, and #75) were selected to evaluate the children’s oral hygiene status by using the Visible Plaque Index (VPI) [20]. The presence of visible plaque on the labial or buccal surfaces of the above teeth were scored as the presence of visible plaque (score of 1) or the absence of visible plaque (score of 0). The percentage of the six index teeth with visible plaque was calculated. The intra-examiner reliability was assessed by randomly re-examining 10% of the recruited children on the same day.

### 2.4. Data Analysis

The statistical analysis was performed by using IBM SPSS Statistics and STATA. Before the data entry, a dental assistant was responsible for checking the returned questionnaires, and the missing data were followed up by phone calls. The intra-examiner reliability was measure by Kappa statistics. Chi-square tests, Mann–Whitney U tests, or Kruskal–Wallis H tests were employed to compare the differences in dental caries experiences among the recruited children when appropriate. The Poisson model, zero-inflated model, negative binomial model and zero-inflated negative binomial (ZINB) regression model were adopted to explore the associations between children’s sociodemographic, oral-health-related behaviors, and dental caries status. Covariates was selected by the backward stepwise method, and the most appropriate model was selected by using the Vuong’s test. The above models estimated the odds ratios (ORs), 95% confidence intervals (CIs), and incidence risk ratios (IRRs). The significance level was set at 0.05.

## 3. Results

### 3.1. Characteristics of the Recruited Children

Four hundred and fifty-two parents signed written consent forms, and all the children received oral examinations (response rate 100%). Of those, 55% (n = 249) were boys. More than half of the parents had received secondary education, and 12% had received primary education or below. Only 2% (n = 2) of the recruited children had begun to brush their teeth before 2 years old, 3% (n = 13) of the children had never used toothpaste, and 13% (n = 59) of the children brushed their teeth less than one time per day. Less than a quarter (n = 98, 22%) of the recruited children had consumed candies every day. In addition, 81% (n = 366) of the children had no dental visit experience during the prior year (Table 1).

### 3.2. Dental Caries and Its Associated Factors among the Recruited Yi Children

The recruited Yi children’s dmft scores were positively skewed. The overall dental caries prevalence among the recruited children was 83%, and 1% of the children had their decayed teeth restored (Table 2). The mean dmft (±Standard Deviation (SD)) score of those children was 5.2 (±4.4), the mean mt (±Standard Deviation (SD)) score was 0.02 (±0.16), and the mean ft (±Standard Deviation (SD)) score was 0.02 (±0.15). Their mean VPI (±Standard Deviation (SD)) score was 57% ± 24%. The intra-examiner and inter-rater reliability for the dmft and VPI were over 0.90.

The bivariate analysis found no differences in dental caries prevalence between sexes (*p* = 0.234) or the median dmft scores (*p* = 0.375). Moreover, neither the children’s dietary habits, tooth-brushing habits, or VPI scores nor their parents’ education attainments were associated with the dental caries experience among the recruited children (*p* > 0.05; Table 3 and Table 4).

However, the children’s dental visit experiences were related to caries experiences. The analysis of bivariate data indicated that caries prevalence was higher among children who had visited a dentist within the prior year than it was for their peers who had no dental visit experience during the prior year (94% vs. 81%, *p* = 0.003; Table 3). Likewise, children who had visited a dentist within the prior year exhibited a higher rank of median dmft score than those peers who had no dental visits exhibited (*p* < 0.001; Table 4).

The results of Vuong’s test demonstrated that the ZINB model could better predict values close to the observed data when compared with other models (*p* < 0.001). Both the ZINB portion and the negative binomial portion indicated that dental visit experience was associated with children’ s dental caries status (IRR = 0.725; 95% CI, 0.600–0.852), whereas children who had not visited a dentist within the prior year were more likely to have caries-free status (OR = 4.959; 95% CI, 1.019–24.142, Table 5).

## 4. Discussion

To date, this was the first study that has investigated dental caries status and its related risk factors among 5-year-old Yi children in the Yunnan province. Four hundred and fifty-two children participated in this cross-sectional study. There was a high level of intra-examiner reliability for the caries assessment (Kappa > 0.90). The overall dental caries prevalence among the recruited children was 83%. The children’s average dmft score was 5.2 (±4.4), while the ft score was 0.02 (±0.15). When children’s oral hygiene status, children’s oral-health-related behaviors, and parents’ education attainments were considered, only children’s dental visit experience was associated with their caries status.

The principal finding of this study revealed that Yi children residing in the Yunnan province had a high prevalence of dental caries (83%). This was much higher than the results of an investigation which was published before 2012 (36–40%) [17,21,22]. Another observational study conducted in Sichuan demonstrated that the dental caries prevalence was lower among Yi preschool children than it was among Han (the majority of the Chinese population) (41.21% vs. 49.19%, *p* < 0.05) [23]. When compared to the prior studies, the caries prevalence was higher in this study, which might partly be due to the sampling selection. The sampling method had not been stated in prior studies which had been performed in the Yunnan province [17,22], whereas the study conducted in Sichuan province only recruited children in a single district by using a convenient sampling method [23]. However, this study adopted a multistage cluster sampling method to recruit 5-year-old Yi children and the representativeness in this study was maintained. The sampling bias in this study might be lower than the mentioned ones. Moreover, the sampling strategy adopted in this study is convenient and cost-effective in terms of participant recruitment, especially for the ethnic minority groups who live in remote and mountainous regions [19]. Additionally, as this study was supported by the Yunnan Education Bureau, all schools were willing to join and help to recruit samples. For children who were absent on the examination day, new appointments were made. The investigators returned to the kindergartens several times until all the children whose parents had signed the consent forms were examined. Thus, this study maintained a high response rate.

It is well recognized that ethnic minority groups have their own traditions and lifestyles, and those may have a significant influence on the occurrence of dental caries status among young children [14]. When comparing the previous studies conducted using the same methods, the caries prevalence and dmft score of Yi preschool children (83%, 5.2 ± 4.4) was similar to Lisu children (80%, 5.6 ± 4.8), as well as Bulang preschool children (85%, 5.8 ± 4.9) [13,14]. However, Dai preschool children showed a higher caries prevalence (89%) and higher dmft score (7.0 ± 5.3) than Yi preschool children did [24]. This was partly due to their dietary habits and living habitations. Living in the southwest part of the subtropical valley areas, sticky rice and sugar cane are popular among Dai people, which can expose young children to a higher risk of dental caries [24]. However, the Yi ethnic minorities are distributed between the southwestern plateau and the southeastern coastal hilly regions of China, which mainly consist of mountains and valleys. Buckwheat, corn, wild herbs, potatoes, beef, mutton, pork, and chicken are popular among Yi people. The dietary habits of Yi children were less cariogenic than those of Dai children. In this investigation, only 22% of the Yi preschool children consumed candies every day, whereas daily snacking was observed among 64% of the Dai preschool children [24].

The relationship between children’s dental caries status and their oral-health-related behaviors was also investigated. The ZINB regression model indicated children’s dental visit experience was associated with their dental caries status, and children who had not visited a dentist within the prior year were more likely to have no dental caries (OR = 4.959; 95% CI, 1.019–24.142). This finding was consistent with the prior studies [13,24,25], demonstrating that preschool children who had dental-visit experience showed higher dmft scores than those children who had never visited a dentist. Although the caries prevalence was high among 5-year-old Yi children, only 1% of the children’s decayed teeth had been treated. Those findings indicated that the Yi children face significant barriers to healthcare. This was partly due to their geographical locations, because most of the ethnic groups, especially Yi people, live in remote, high-altitude areas [26]. Some of those regions are inaccessible by public transportation, and insufficient healthcare resources are common in those regions. Moreover, the Yunnan province has an acute shortage of health personnel resources. According to the data set pooled from 2011 [27], the number of healthcare providers in Yunnan was 215,335, whereas it was 374,157 in Zhejiang, which was a developed province in China. The treatment needs of 40,886 patients had been met in the Zhejiang province, whereas only 18,024 patients have received expected treatment in the Yunnan province. In terms of the ethnic minority areas in the Yunnan province, the situation was much worse. Ethnic minorities mainly inhabit less-developed rural areas. However, among the 215,335 healthcare providers in the Yunnan province, only 35,672 (16.57%) were in rural areas [27]. In addition, the GDP per capita of the Yi Autonomous Prefecture was lower than the average provincial GDP per capita [15]. Due to the economic burden, patients in those areas were less likely to seek timely treatment. In further studies, oral health promotion activities are encouraged to release the burden of inadequate dental services among Yi preschool children.

Although there was evidence that children’s caries status was also related to poor oral hygiene status, inappropriate snacking habits, unfavorable oral hygiene practice, and family socio-economic status [13,14], we found no statistically significant relationship of those factors with dental caries. As cross-sectional studies have their limitations, the causal effect cannot be estimated. Further studies with a larger cohort are warranted to distinguish the potential factors which may play an essential role in the progression of dental caries among those children.

## 5. Conclusions

The dental caries prevalence among 5-year-old Yi children in the Yunnan province was high, whereas few of the cavities had been restored. Yi children’s dental caries status was related to their dental visit experience.

## Figures and Tables

**Table 1:** Characteristics of recruited preschool children (n = 452).

Variables	n (%)
***Background Information***	
Sex	
Boys	249 (55)
Girls	203 (45)
Father’s education level	
Primary or below	52 (12)
Secondary	288 (64)
Tertiary or above	112 (25)
Mother’s education level	
Primary or below	55 (12)
Secondary	303 (67)
Tertiary or above	94 (21)
***Oral-health-related behaviors***	
Frequency of candy intake (times/day)	
<1	354 (78)
≥1	98 (22)
Frequency of sour snacks intake (times/day)	
<1	395 (87)
≥1	57 (13)
Age at which tooth brushing began (months)	
<24	9 (2)
≥24	443 (98)
Brushing frequency (times/day)	
<1	59 (13)
≥1	393 (87)
Brushing with toothpaste	
Yes	439 (97)
No	13 (3)
Visited a dentist within last year	
Yes	86 (19)
No	366 (81)
**VPI**	
score ≤ 50%	241 (53)
score > 50%	211 (47)

**Table 2:** Caries experience (dmft) of the Yi children (n = 452).

Dental Caries	Prevalence	Mean (SD)
Caries experience (dmft)	83%	5.2 (4.4)
Decay teeth (dt)	83%	5.1 (4.4)
Missing teeth (mt)	1%	0.02 (0.16)
Filled teeth (ft)	1%	0.02 (0.15)

**Table 3:** Caries prevalence (dmft > 0) and independent variables (n = 452) (Chi-square test).

Variables	dmft > 0	*p*-Value
***Background Information***		
Sex		0.234
Boys	82%	
Girls	86%	
Father’s education level		0.512
Primary or below	89%	
Secondary	83%	
Tertiary or above	81%	
Mother’s education level		0.752
Primary or below	84%	
Secondary	84%	
Tertiary or above	81%	
***Oral-health-related behaviors***		
Frequency of candy intake		
<1	84%	0.821
≥1	83%	
Frequency of sour snacks intake		0.836
<1	84%	
≥1	83%	
Age at which tooth brushing began		0.177
<24 m	100%	
≥24 m	83%	
Brushing frequency		0.65
<1	81%	
≥1	84%	
Brushing with toothpaste		0.905
Yes	83%	
No	85%	
Dental visit within last year		0.003
Yes	94%	
No	81%	
**VPI**		0.61
score ≤ 50%	83%	
score > 50%	84%	

**Table 4:** Median dmft scores and independent variables (n = 452) (Mann–Whitney U tests or Kruskal–Wallis H tests).

Variables	Median (Q1, Q3)	Rank of Median	*p*-Value
***Background information***			
Sex			0.375
Boys	1 (4, 7.5)	222	
Girls	2 (4, 8)	233	
Father’s education level			0.454
Primary or below	2 (6, 8.75)	247	
Secondary	2 (4, 8)	225	
Tertiary or above	1.25 (4, 8)	220	
Mother’s education level			0.551
Primary or below	2 (5, 10)	241	
Secondary	2 (4, 8)	227	
Tertiary or above	1 (4, 8)	217	
***Oral-health-related behaviors***			
Frequency of candy intake			0.684
<1	2 (4, 8)	225	
≥1	1 (5, 8.75)	231	
Frequency of sour snacks intake			0.472
<1	2 (4, 8)	224	
≥1	2 (5, 8.5)	238	
Age at which tooth brushing began			0.681
<24 m	2 (3, 9)	244	
≥24 m	2 (4, 8)	226	
Brushing frequency			0.325
<1	1 (4, 7)	210	
≥1	2 (4, 8)	228	
Brushing with toothpaste			0.9
Yes	2 (4, 8)	226	
No	1.25 (5, 8)	231	
Dental visit within last year			<0.001
Yes	4 (7.5, 11)	296	
No	1 (4, 7)	210	
**VPI**			0.675
score ≤ 50%	1.5 (4, 8)	224	
score > 50%	2 (5, 8)	229	

**Table 5:** Caries risk factors of the study children (ZINB regression).

**Zero-Inflated Portion (dmft = 0)**	**Variables**	**OR**	**95% CI**		***p*-Value**
	**Dental Attendance**				
	Yes ^a^				
	No	4.959	1.019	24.143	0.047
**Negative Binomial Portion (dmft > 0)**	**Variables**	**IRR**	**95% CI**		***p*-Value**
	**Dental attendance**				
	Yes ^a^				
	No	0.725	0.600	0.852	<0.01

^a^ Reference group; OR = odds ratio, IRR = incidence risk ratio; CI = confidence interval.

## Data Availability

The datasets generated and/or analyzed during the current study are available from the corresponding author upon reasonable request.
